# Indented Tongue

**Published:** 1888-08

**Authors:** 


Indented Tongue.—In answer to inquiries sometimes addressed to us to name remedies having this symptom, we give the following: Ars-met., Glon., Hydrast., Ign., Iod., Kali-jod., Merc., Podophyl., Rhus-t., Stram., Tellur-ac. Most of these remedies were given to us by the venerable Dr. Lippe.

